# The Concept of Equivalent Radon Concentration for Practical Consideration of Indoor Exposure to Thoron

**DOI:** 10.3390/ijerph9010286

**Published:** 2012-01-18

**Authors:** Jing Chen, Deborah Moir

**Affiliations:** Radiation Protection Bureau, Health Canada, 2720 Riverside Drive, Ottawa K1A 0K9, Canada; Email: deborah.moir@hc-sc.gc.ca

**Keywords:** radon-220, thoron, radon-222, radon, indoor exposure

## Abstract

To consider the total exposure to indoor radon and thoron, a concept of equivalent radon concentration for thoron is introduced, defined as the radon concentration that delivers the same annual effective dose as that resulting from the thoron concentration. The total indoor exposure to radon and thoron is then the sum of the radon concentration and the equivalent radon concentration for thoron. The total exposure should be compared to the radon guideline value, and if it exceeds the guideline value, appropriate remedial action is required. With this concept, a separate guideline for indoor thoron exposure is not necessary. For homes already tested for radon with radon detectors, Health Canada’s recommendation of a 3-month radon test performed during the fall/winter heating season not only ensures a conservative estimate of the annual average radon concentration but also covers well any potentially missing contribution from thoron exposure. In addition, because the thoron concentration is much lower than the radon concentration in most homes in Canada, there is no real need to re-test homes for thoron.

## 1. Introduction

Radon is a naturally occurring radioactive gas generated by the decay of uranium and thorium bearing minerals in rocks and soils. Radon and its decay products are the major contributors to human exposure from natural radiation sources [1,2] and have been identified as the second leading cause of lung cancer after tobacco smoking [3,4]. Radon-222 and radon-220 are the most common isotopes of radon, with the term “radon” typically referring to radon-222 and “thoron” to radon-220. These naming conventions will be used in this publication.

Based on new scientific information and a broad public consultation, the Canadian radon guideline was lowered from 800 to 200 Bq·m^−3^ in June 2007 [5]. To support the implementation of the revised guideline, a National Radon Program was developed in collaboration with the Federal Provincial Territorial Radiation Protection Committee, an intergovernmental committee established to advance the development and harmonization of practices and standards for radiation protection within the various Canadian jurisdictions. Significant progress has been made in the National Radon Program since its inception in 2007. As part of the Program, the Government of Canada initiated a project to test radon levels in federal buildings in order to minimize health risks to employees and the public frequenting those buildings. A national radon education and awareness strategy was commenced in 2008. The strategy is focused on raising awareness of radon, the potential health risks from exposure to elevated indoor radon levels and encouraging Canadians to test their homes and to reduce radon levels where necessary. To gain a better understanding of radon concentrations in homes across Canada, a national residential radon survey was launched in April 2009. The survey uses alpha track detectors deployed for a minimum of three months during the heating season (Oct-April) with the objective of testing about 18,000 homes over a two year period [6]. More and more Canadians are becoming aware of the health risks associated with radon exposure and are testing their homes for radon.

In recent years, exposure to thoron and its possible health effects have gained increasing attention as indicated in a recent report of the United Nations Scientific Committee on the Effects of Atomic Radiation (UNSCEAR) [2]. Research on thoron and its contribution to the total radiation exposure from radon isotopes (both radon-222 and radon-220) has been carried out since the beginning of the National Radon Program in 2007. Results of several small scale Canadian thoron surveys have been published [7,8,9,10]. It is estimated that thoron contributes about 8% of the radiation dose attributed to indoor exposure to both radon-222 and radon-220 in Canada. Indoor thoron exposure is now becoming an issue of health interest to Canadians. More frequently, questions related to thoron are arising from the public; what are the differences between radon and thoron? Do we need to re-test our homes for radon because of the existence of thoron? Is it necessary to have a new guideline for indoor thoron? This paper tries to address the questions related to radon/thoron testing and the need for a thoron guideline with the proposed concept of using an equivalent radon concentration to express thoron levels in homes.

Radon (radon-222) has a half-life of 3.82 days and originates from the uranium (uranium-238) decay chain. The half-life of thoron (radon-220), a product of the thorium (thorium-232) decay series, is much shorter at 55.6 s. As a result of its short half-life, thoron infiltration from the ground and subsequent migration from its production site to the immediate environment of people within a building is limited. Thus, while radon tends to be homogeneously distributed in indoor air, the thoron level varies significantly with distance from the source. One of the thoron decay products, lead-212, is relatively long-lived (10.6 h) allowing it time to migrate into the immediate environment of building occupants before further decaying to produce the alpha-emitting isotope bismuth-212 (with a half-life of 60.6 min). Therefore, unlike thoron gas, thoron decay products tend to be more homogeneously distributed in indoor air. When radon or thoron and their decay products are inhaled into the lungs, the mechanism by which they produce damage is the same, *i.e.*, alpha emission causing ionization within lung tissue which can lead to lung cancer. The risk is proportional to the effective doses associated with radon and thoron. As estimated by the UNSCEAR 2006 Report [2], thoron contributes on average about 10% of the total exposure to indoor radon and thoron. However, the variation is large for various geographical locations and there are situations where exposure to thoron is much more significant. Simultaneous radon and thoron measurements were conducted in a total of 370 Canadian homes in five cities/communities [7,8,9,10]; the ratios between thoron and radon concentrations in individual homes varied widely with an average ratio of 0.67. Three point eight percent (3.8%) of homes had thoron concentrations more than five times the radon concentration. Therefore, exposure to indoor thoron cannot be ignored in a national radon program.

## 2. The Concept of Equivalent Radon Concentration for Thoron

The annual effective dose due to indoor radon exposure, *E*_Rn_, was assessed based on the formula given by the UNSCEAR report [1]:


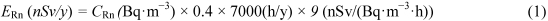


where *C*_Rn_ is the arithmetic mean radon concentration in the units of Bq·m^−3^, the typical value of 0.4 was used as the equilibrium factor for radon indoors, a recommended value of 9 nSv (Bq·m^−3^ h)^−1 ^was used to convert radon equilibrium-equivalent concentration (EEC) to the effective dose, and an 80% home occupancy time, *i.e.*, 7000 h per year, was assumed.

The annual effective dose due to indoor thoron exposure, *E*_Tn_, was assessed based on the formula [1]:


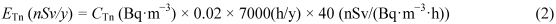


where *C*_Tn_ is the arithmetic mean thoron concentration in the units of Bq·m^−3^, the typical value of 0.02 was used as the equilibrium factor for thoron indoors, a recommended value of 40 nSv (Bq·m^−3^h)^−1 ^was used to convert thoron EEC to the effective dose.

In the above conversion of indoor thoron exposure to the annual effective dose, the typical value of 0.02 was used for the equilibrium factor *F*. As indicated in the UNSCEAR 2006 Report [2], the equilibrium factor between thoron gas and its decay products was not well established. The recommended typical value of 0.02 should be regarded as being subject to large uncertainties. Long-term simultaneous thoron gas and its progeny measurements in a total of 113 Canadian homes [11] showed that the equilibrium factor is log-normally distributed with a geometric mean of 0.022 and a geometric standard deviation of 3.02. The results indicate that the *F* value of 0.02 for indoor thoron recommended by UNSCEAR is a reasonable value for those Canadian homes tested.

The equivalent radon concentration, *R*_Tn_, for an exposure to the thoron concentration (*C*_Tn_), is the radon concentration that delivers the same annual effective dose as resulted from *C*_Tn_:


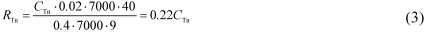


A total of 370 Canadian homes have been tested for thoron in addition to radon [7,8,9,10] with thoron detected in about half of those. Among the homes with detectable thoron concentrations, the values varied from 6 to 1977 Bq·m^−3^ with a population weighted average of 56 Bq·m^−3^. Table 1 provides a look-up table for easy conversion between thoron concentrations found in Canadian homes and the corresponding equivalent radon concentrations. One can see from Equation 3, or using Table 1 to estimate, the average exposure to indoor thoron of 56 Bq·m^−3^ corresponds to an equivalent radon exposure of 12 Bq·m^−3^.

**Table 1:** Thoron concentrations, *C_Tn_*, and their corresponding equivalent radon concentrations, *R_Tn_*.

*C*_Tn_ (Bq m^−3^)	*R*_Tn_ (Bq m^−3^)	*C*_Tn_ (Bq m^−3^)	*R*_Tn_ (Bq m^−3^)
10	2	200	44
20	4	400	88
40	9	600	132
60	13	800	176
80	18	1000	220
100	22	2000	440

## 3. Discussion

The total indoor exposure to radon and thoron could be regarded as the sum of the radon concentration and the equivalent radon concentration for thoron. This total exposure can then be compared to the radon guideline value. If it exceeds the guideline value of 200 Bq·m^−3^, appropriate action is required to reduce the risk. In this way, a separate guideline for indoor thoron exposure would not be necessary.

For radon testing, long-term measurements are recommended by Health Canada. Alpha track detectors (ATDs) are commonly used for this purpose. There are various types of ATDs available commercially, some sensitive to thoron and some to radon only [12]. There are a few ATDs capable of measuring radon and thoron separately, such as the RADUET used in the simultaneous radon and thoron surveys in Canada [7,8,9,10].

If an ATD is designed for radon measurement, but sensitive to thoron, it gives a total concentration estimate:


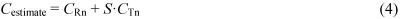


where S is the fractional thoron sensitivity. Depending on the design of ATDs, *S* can vary from less than 0.01 to as large as 0.90 (*i.e.*, 90%) [12]. From the above definition of the equivalent radonconcentration, a correct estimate should be the total exposure concentration in the sense of the effective dose:


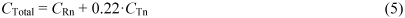


If an ATD has a sensitivity *S* < 0.22, the total exposure concentration will be underestimated. An overestimation of the total exposure concentration would occur when *S* > 0.22.

An over-estimation (*S* > 0.22) of the total exposure could result in “unnecessary” action if *C*_estimate _> 200 Bq m^−3^. However, while this may at first appear to be a drawback, it is in reality a proactive action. Among six types of passive radon detectors used in past national radon surveys in Canada, Germany, Ireland, Italy, the United Kingdom, and the USA, only two detector types have a high sensitivity for thoron, *S* = 0.78 for a German detector type and *S* = 0.68 for one of the detector types made in the USA [12]. If a radon detector with *S* = 0.8 were used for radon testing, there will only be a slight overestimation of the total exposure, because thoron concentration is only a fraction of the radon concentration in the majority of homes (less than 40% of the radon concentration in more than 70% of homes).

Most of the radon detectors used in past national radon surveys have a small sensitivity to thoron [12], *S* ≤ 0.05. For ATDs with thoron sensitivity significantly less than 0.22 (*S* << 0.22), such as the ATDs used in the Winnipeg case-control study in the 1980s (*S* = 0.02) [13], the total exposure could be underestimated. However, as estimated by the UNSCEAR 2006 Report [2], thoron contributes about 10% of the total exposure to indoor radon and thoron worldwide. Canadian studies [7,8,9,10] confirmed that thoron could contribute about 8% of the total indoor exposure to radon and thoron. Therefore, on average, the total exposure from those early survey results could be underestimated by about 8–10%.

As there is some risk at any radon level, it is important to avoid underestimation of the radon level. Health Canada’s recommendation of a 3-month radon test performed during the fall/winter heating season [14] results in an estimated radon concentration, on average, about 20% higher than the value determined from a one-year measurement [15,16]. This measurement protocol not only ensures a conservative estimate of the annual average radon concentration but also would compensate for any potentially missing contribution from thoron exposure.

It should be noted that the 10−20% under- or overestimation on radon concentration by typical radon detectors is within the measurement uncertainty of these detectors and within the radon fluctuation in individual homes from year to year. A recent intercomparison [17] of radon detectors, in which 26 organizations from 16 countries participated, demonstrated that 69% of the results fell within 20% from the reference value of the radon concentration. In other words, more than 30% of radon detectors provided an estimate of the radon concentration ± 20% away from the true radon level. It is a well known fact that radon concentration inside a home varies over time. It is not uncommon to see radon levels in a house change by a factor of two or more over a one-day period. Seasonal variations and changes year-by-year were frequently observed. A study of annual average indoor radon variations over two decades at 196 sites in 98 Minnesota houses [18] showed that year-to-year radon variations at individual sites ranged from 3 to 110% with the median variation of 26%. Therefore, generally speaking, provided Health Canada’s recommended protocol for radon testing was used, there is no real need to re-test homes in order to obtain such a small potential additional dose (~10%) from exposure to indoor thoron.

## 4. Conclusions

Recent attention to the issue of thoron exposure in homes has raised questions about the need for a separate thoron guideline similar to the Canadian Radon Guideline. In addition, homeowners are concerned that if they have already tested their homes for radon, they may need to carry out a second, separate test to determine their risk from thoron exposure.

We have shown that the first issue can be circumvented by using the concept of an equivalent radon concentration for exposure to thoron, *i.e.*, the radon concentration that delivers the same annual effective dose as from a given thoron concentration. The total indoor exposure to radon and thoron is the sum of the radon concentration and this equivalent radon concentration for thoron. A separate guideline for indoor thoron exposure is therefore not necessary.

However, to apply this concept both the radon and thoron concentrations in a home are needed which requires the use of detectors capable of discriminating between radon and thoron. Many homes have already been tested for radon with radon detectors only, and thoron discriminating detectors are not readily available.

Health Canada’s recommendation of a 3-month radon test performed during the fall/winter heating season ensures a conservative estimate of the annual average radon concentration, as it tends to give a slight over-estimation (~20%) of the radon concentration in a home. If the detectors used are not significantly sensitive to thoron, the margin of over-estimation provided by the testing protocol has the potential to cover any missing contribution from thoron exposure. In general, thoron concentrations in most Canadian homes have been found to be much smaller than radon concentrations. This suggests there is no real need to re-test homes for thoron to factor in such a small additional contribution to the overall exposure, especially as it is largely compensated for in the testing protocol. However, radon detectors having a high sensitivity to thoron could result in a high false positive for radon, causing undue concern for homeowners. Health Canada is currently conducting a study on thoron sensitivity for all commercially available radon detectors in the Canadian market.

## Conflict of Interest

The authors declare no conflict of interest. 
